# Supplementary material for: Decisional Conflict after Deciding on Potential Participation in Early Phase Clinical Cancer Trials: Dependent on Global Health Status, Satisfaction with Communication, and Timing
Source: Cancers (Basel). 2022 Mar 15;14(6):1500. doi: 10.3390/cancers14061500 (PMC8946532; doi:10.3390/cancers14061500)
Supplement: Supplementary file 1 [file cancers-14-01500-s001.zip › Table S1 - Table of (cor)relations.pdf]

Table S1. Table of (cor)relations

	Decisional conflict	Global health status	Physical functioning	Role functioning	Emotional functioning	Cognitive functioning	Social functioning	Fatigue	Nausea and vomiting	Pain	Dyspnoea	Insomnia	Appetite loss	Constipation	Diarrhoea	Financial difficulties	Health literacy	Sense of Hope	Satisfaction	Decision‡
**Global health status**	-.322**																			
**Physical functioning**	-.181	.602**																		
**Role functioning**	-.151	.507**	.548**																	
**Emotional functioning**	-.122	.420**	.275**	.364**																
**Cognitive functioning**	-.083	.303**	.379**	.387**	.237*															
**Social functioning**	-.200*	.537**	.430**	.471**	.503**	.455**														
**Fatigue**	-.208*	-.616**	-.576**	-.578**	-.453**	-.421**	-.456**													
**Nausea and vomiting**	-.021	-.178	-.183*	-.158	-.201*	-.044	-.126	.365**												
**Pain**	.193*	-.602**	-.489**	-.478**	-.313**	-.285**	-.480**	.508**	.219*											
**Dyspnoea**	.165	-.206*	-.404**	-.325**	-.168	-.182	-.231*	.351**	.265**	.101										
**Insomnia**	.154	-.234*	-.182	-.212*	-.263**	-.274**	-.284**	.406**	.141	.353**	.117									
**Appetite loss**	.042	-.266**	-.177	-.250**	-.360**	-.095	-.175	.476**	.277**	.191*	.142	.216*								
**Constipation**	.214*	-.301**	-.177	-.194*	-.184*	-.080	-.166	.297**	.285**	.308**	.168	.196*	.189*							
**Diarrhoea**	.015	.045	.050	-.073	.113	.023	-.042	.160	.363**	-.029	.114	.017	.309**	.178						
**Financial difficulties**	.090	-.063	-.112	-.169	-.014	-.119	-.205*	.087	.087	.086	.186*	.012	.059	.055	-.018					
**Health literacy**	-.211*	.255**	.138	.205*	.392**	.190*	.128	-.133	-.074	-.107	.013	-.117	-.085	-.055	.039	-.017				
**Hope**	-.278**	.442**	.175	.346**	.372**	.192*	.245**	-.334**	-.041	-.281**	-.109	-.030	-.191*	-.117	.078	-.124	.212*			
**Satisfaction**	-.387**	.088	.018	.068	.260**	-.044	.108	-.080	.043	-.060	-.138	.001	-.043	-.066	.099	-.189*	-.057	.107		
**Decision⊥**	-2.127*	.868	.008	.221	1.785	.738	.016	.004	5.346*	.046	.580	.383	.112	5.089*	3.493	.292	11.354**	2.086	5.601*	
**Timing†**	16.135**	2.825*	1.549	2.731*	2.023	2.169	3.650*	1.767	.549	1.976	3.879*	1.023	1.300	2.860*	.324	.692	.988	1.709	3.108*	7.087

* = p<0.05; ** = p<0.01

⊥= values for decision * continuous variables from t-tests; †= values for timing * continuous variables from ANOVAs; ‡= value for decision * timing from Fisher's exact test
