# Supplementary figures and images for: Decisional Conflict after Deciding on Potential Participation in Early Phase Clinical Cancer Trials: Dependent on Global Health Status, Satisfaction with Communication, and Timing
Source: Cancers (Basel). 2022 Mar 15;14(6):1500. doi: 10.3390/cancers14061500 (PMC8946532; doi:10.3390/cancers14061500)

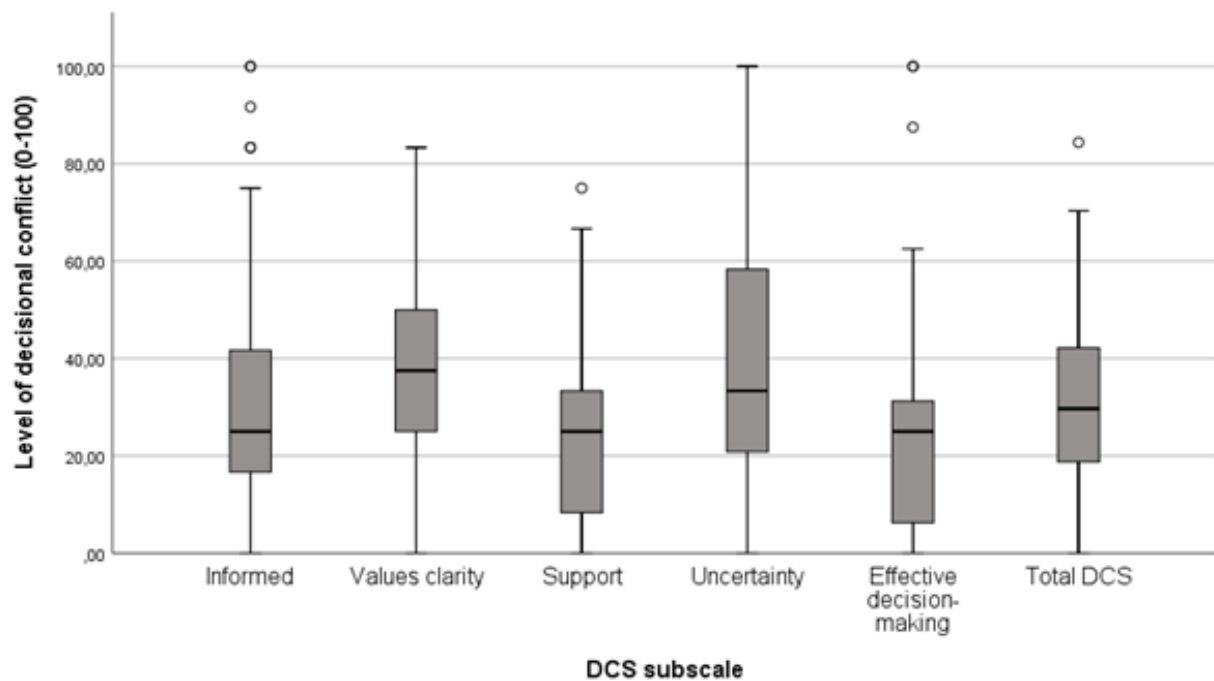

Supplement: Supplementary file 1 [file cancers-14-01500-s001.zip › Figure S1 - Boxplots DCS.pdf]

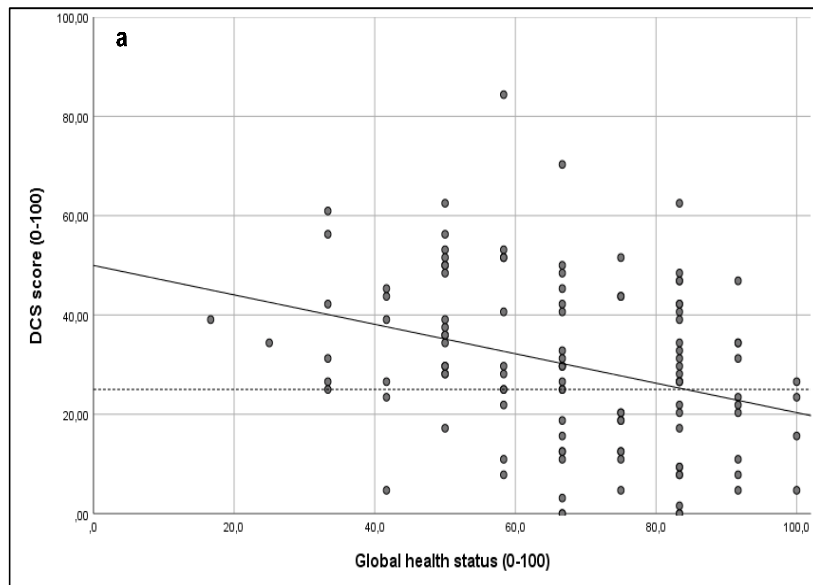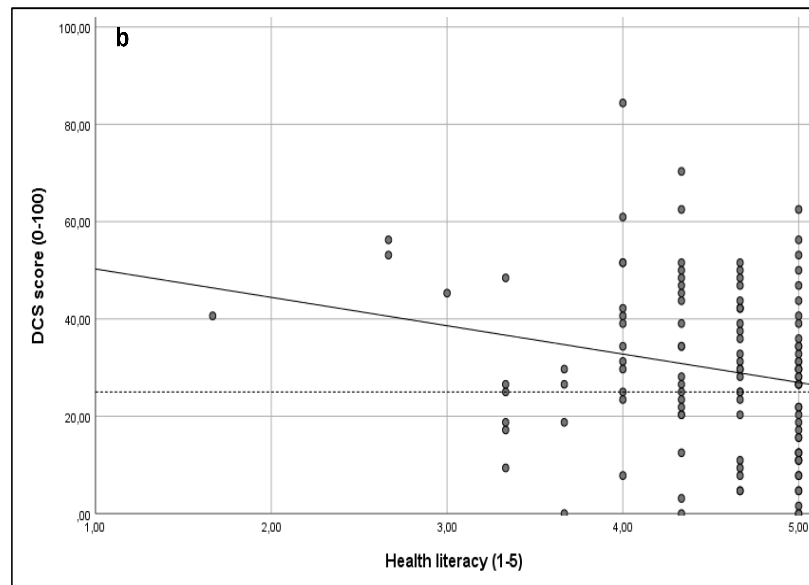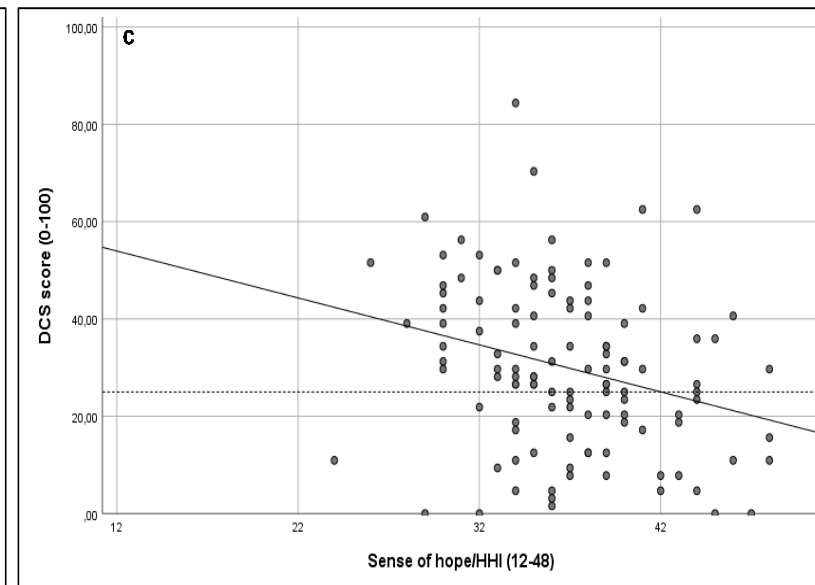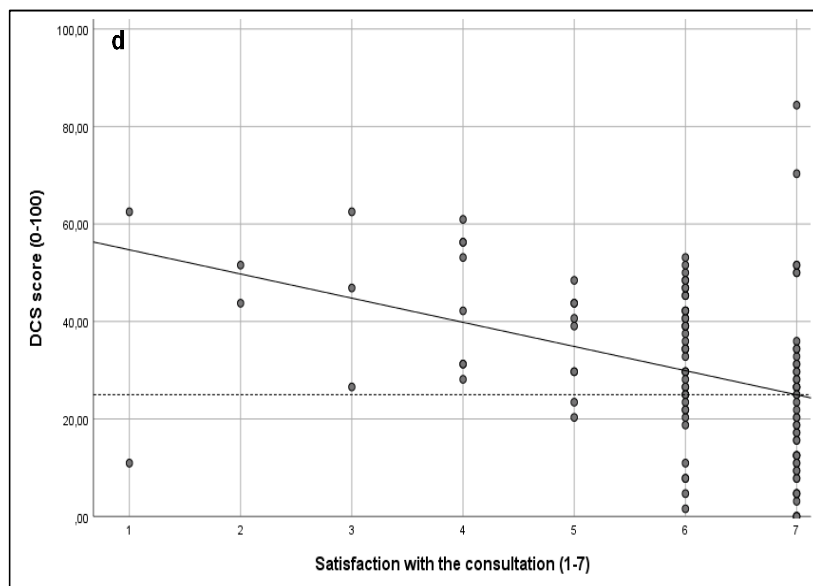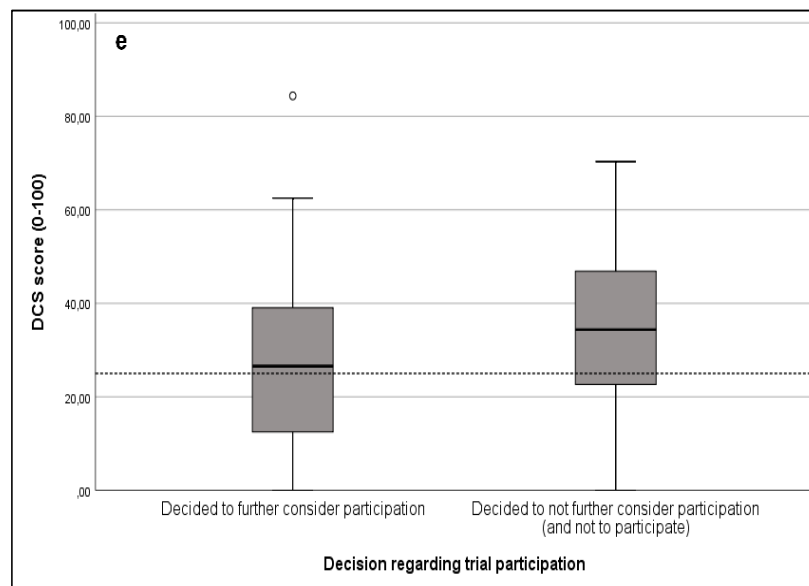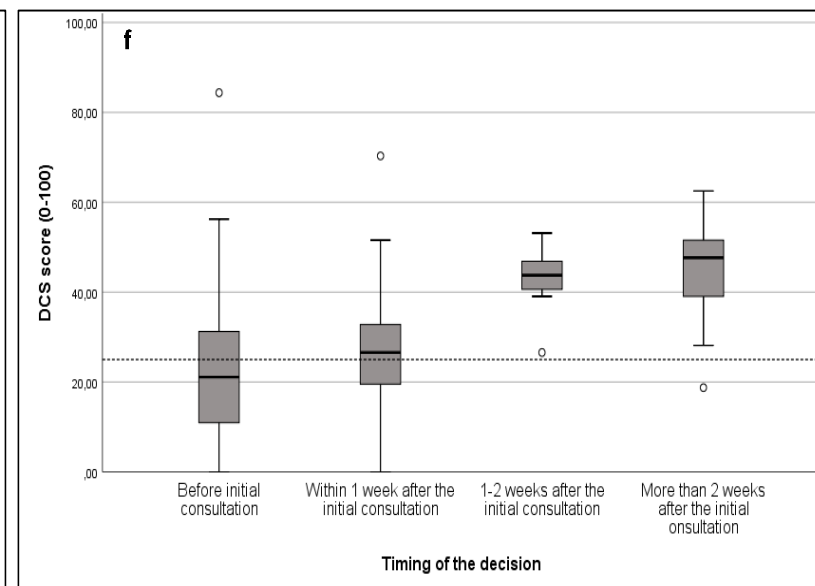

Supplement: Supplementary file 1 [file cancers-14-01500-s001.zip › Figure S2 - Histograms and boxplots.pdf]
